# Association of mutation and expression of the brother of the regulator of imprinted sites (BORIS) gene with breast cancer progression

**DOI:** 10.18632/oncotarget.28442

**Published:** 2023-05-26

**Authors:** Mohammad Salman Akhtar, Naseem Akhter, Arshi Talat, Raed A. Alharbi, Abdulmajeed A.A. Sindi, Faisal Klufah, Hanan E. Alyahyawi, Abdulmohsen Alruwetei, Abrar Ahmad, Mazin A. Zamzami, SVS Deo, Syed Akhtar Husain, Osama A. Badi, Mohammad Jahir Khan

**Affiliations:** ^1^Department of Basic Medical Sciences, Faculty of Applied Medical Sciences, Al-Baha University, Al-Baha, Saudi Arabia; ^2^Department of Biosciences, Faculty of Natural Sciences, Jamia Millia Islamia, New Delhi, India; ^3^Department of Orthodontics and Dentofacial Orthopedics, ITS Dental College, Hospital and Research Centre, Greater Noida, Delhi-NCR, India; ^4^Department of Laboratory Medicine, Faculty of Applied Medical Sciences, Al-Baha University, Al-Baha, Saudi Arabia; ^5^Department of Medical Laboratory, College of Applied Medical Sciences, Qassim University, Qassim, Saudi Arabia; ^6^Department of Biochemistry, Faculty of Science, King Abdulaziz University, Jeddah, Saudi Arabia; ^7^Department of Surgical Oncology, BRA- IRCH, All India Institute of Medical Sciences (AIIMS), New Delhi, India; ^8^Department of Neurology, Henry Ford Health System, Detroit, MI 48202, USA; ^9^School of Biotechnology, Jawahar Lal Nehru University, New Delhi, India

**Keywords:** BORIS, breast cancer, mutation, transcription factor, PCR-SSCP

## Abstract

Introduction: The BORIS, 11 zinc-finger transcription factors, is a member of the cancer-testis antigen (CTA) family. It is mapped to chromosome number 20q13.2 and this region is genetically linked to the early onset of breast cancer. The current study analyzed the correlation between BORIS mutations and the expression of the protein in breast cancer cases.

Materials and Methods: A population-based study including a total of 155 breast cancer tissue samples and an equal number of normal adjacent tissues from Indian female breast cancer patients was carried out. Mutations of the BORIS gene were detected by polymerase chain reaction-single standard confirmation polymorphisms (PCR-SSCP) and automated DNA sequencing and by immunohistochemistry for BORIS protein expression were performed. The observed findings were correlated with several clinicopathological parameters to find out the clinical relevance of associations.

Results: Of all the cases 16.12% (25/155) showed mutations in the BORIS gene. The observed mutations present on codon 329 are missense, leading to Val> Ile (G>A) change on exon 5 of the BORIS gene. A significant association was observed between mutations of the BORIS gene and some clinicopathological features like nodal status (*p* = 0.013), estrogen receptor (ER) expression (*p* = 0.008), progesterone receptor (PR) expression (*p* = 0.039), clinical stage (*p* = 0.010) and menopausal status (*p* = 0.023). The protein expression analysis showed 20.64% (32/155) samples showing low or no expression (+), 34.19% (53/155) with moderate expression (++), and 45.17% (70/155) showing high expression (+++) of BORIS protein. A significant association was observed between the expression of BORIS protein and clinicopathological features like clinical stage (*p* = 0.013), nodal status (*p* = 0.049), ER expression (*p* = 0.039), and PR expression (*p* = 0.027). When mutation and protein expression were correlated in combination with clinicopathological parameters a significant association was observed in the category of high (+++) level of BORIS protein expression (*p* = 0.017).

Conclusion: The BORIS mutations and high protein expression occur frequently in carcinoma of the breast suggesting their association with the onset and progression of breast carcinoma. Further, the BORIS has the potential to be used as a biomarker.

## INTRODUCTION

Breast cancer is a multifactorial complex heterogeneous disease, and represents about one-fourth of the total cancers diagnosed in females [1, 2]. The incidence of breast cancer from 2012 to 2018 shows a yearly increase of almost 6% (from 1655589 to 2069792) [3]. However, in future estimates annually a reduction of about 50% in breast cancer cases from 2018 to 2040 [4, 5]. About 5% of cases of breast cancer occur because of germline mutations in BRCA1 and BRCA2 [6] and 95% remaining depend on the genetic changes in the breast cancer susceptibility gene. It is associated with various risk factors [7] like genetic and epigenetic changes [8] and various environmental factors.

Brother of the Regulator of Imprinted Sites (BORIS) is the known paralogue of CCCTC-binding factor (CTCF) - a multifunctional DNA binding protein, that uses different sets of zinc fingers to mediate distinct functions in the regulation of gene expression [9, 10]. The BORIS gene is classified as one of the cancer-testis antigen (CTA) family members; expressed during spermatogenesis in the testis [11, 12] as well as in various cancers, like uterine, lung, gastric and cancers of the breast [13–16]. It is indicated that BORIS can act as a biomarker of breast cancer in women for Indian patients as well as breast cancer patients across the world. In breast cancer patients high levels of BORIS protein are detected in leukocytes, suggesting that BORIS can be used as an important biomarker for breast cancer progression [17] and is a male system-specific protein having the same 11-zinc finger structure as that of CTCF [18]. The expression of BORIS suppresses DNA damage and promotes resistance of cisplatin by enhancement of the mismatch repair system of cancer cells [19]. In contrast to CTCF’s tumor suppressive functions, an oncogenic role of the BORIS gene was also reported [11, 20–24]. The upregulation of CTCF helps in the protection of cells from the apoptotic process [25, 26]. However, induced expression levels of BORIS coincided with the progression of tumors, and silenced BORIS expression associated with the progression of apoptosis, it also plays an important role in the regulation of transcription [22, 23, 27–29]. The mechanisms of alteration of BORIS transcription are based on BORIS’ ability to bind the DNA at specific binding motifs [30–32].

CTCF emerged by duplication of the gene during the evolution of amniotes [33] that in human cancer is being proposed to act like an oncogene by dysregulation of cancer epigenome. The gene products of CTA are immunogenic in cancer patients showing high level of restricted expression in tissues [34, 35]. In the process of spermatogenesis, they play an important role by regulating the expression of pluripotency and testis-specific genes [32, 36, 37]. It is also activated in cancers aberrantly of various lineages like lung [38–40] breast [13, 17], uterine [15], esophageal [41], hepatocellular [42], ovarian [43–46], prostate [47], urogenital [48], and neuroblastoma [49]. The neoplastic transformations are promoted by its interference in the cellular processes like invasion, apoptosis, cell proliferation, and immortalization [43, 44, 49–51]. Therefore, it was identified that CTCFL is one of the most promising CTA by the NCI [52], and is a known activator of expression of other various cancer testis antigens.

The BORIS gene is localized on chromosome 20q13.2 [11], in various cancers, this part of the chromosome is amplified and is mainly dominated by immortalizing or transforming genes [53, 54]. Therefore, CTCF-IBORIS-binding of BORIS methylation events is implicated in control regions of imprinting sites [55], and is significant in cancer progression. Normally, the BORIS gene is not expressed in females, their presence can be probed in patients with breast tumors, having characteristic properties of cancer biomarkers that would also be investigated.

The present study is to find out the mutations of BORIS genes in hot spot exons by PCR-SSCP and by automated DNA sequencing in breast cancer tissue samples along with adjacent normal samples. The BORIS protein expression was conducted by immunohistochemical analysis to identify the BORIS mutations in breast cancer progression and to evaluate the relationship between BORIS mutations corresponding to their protein expression with various clinicopathological variables of breast cancer patients.

## RESULTS

### Mutations in BORIS and clinico-pathological parameters

**Figure 1:**
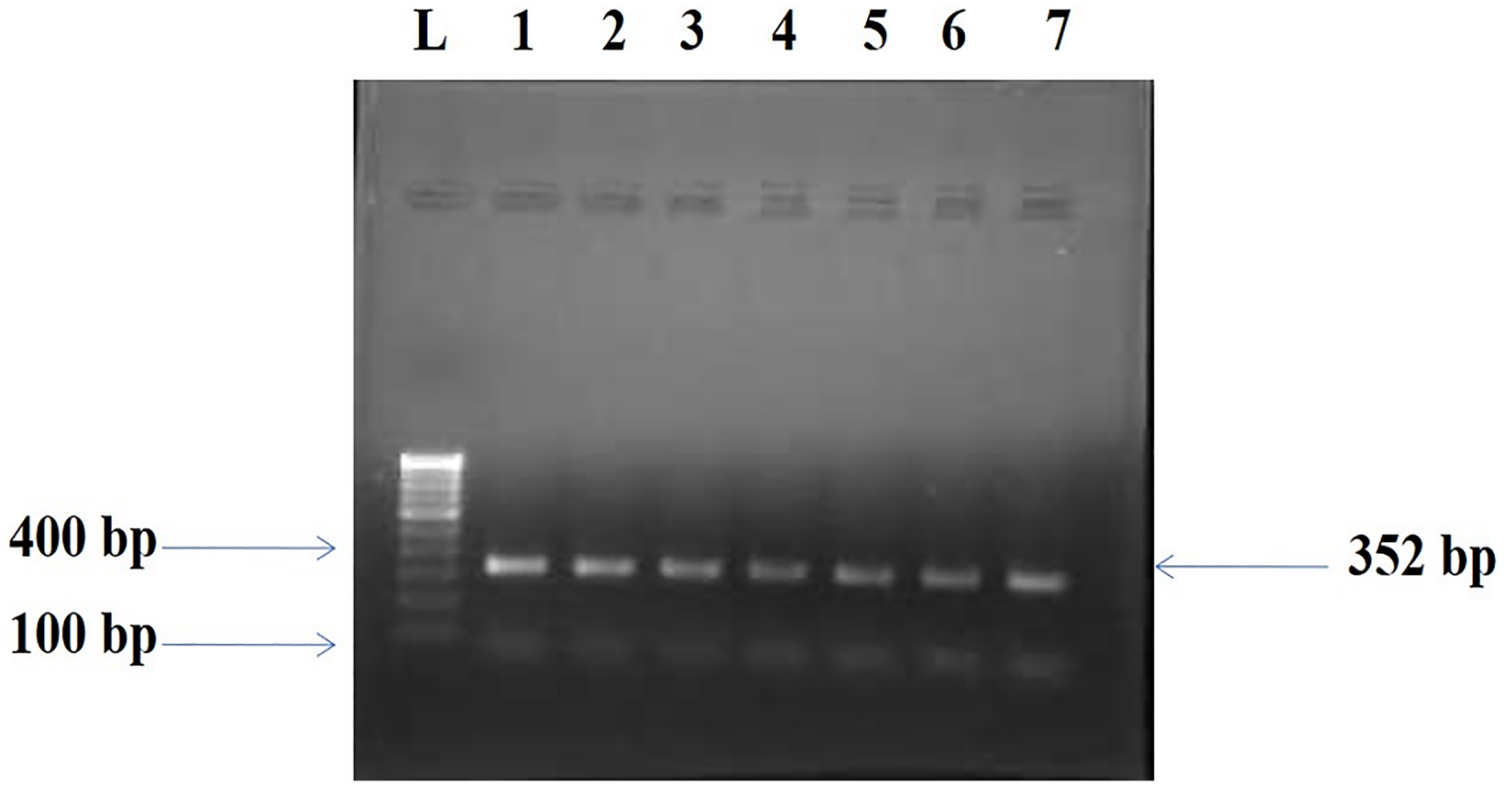
Amplified PCR product 352 bp of BORIS gene. Lane L: Molecular marker of 100 bp, Lanes 1–7: Amplicons from the Breast cancer tissue samples.

**Figure 2:**
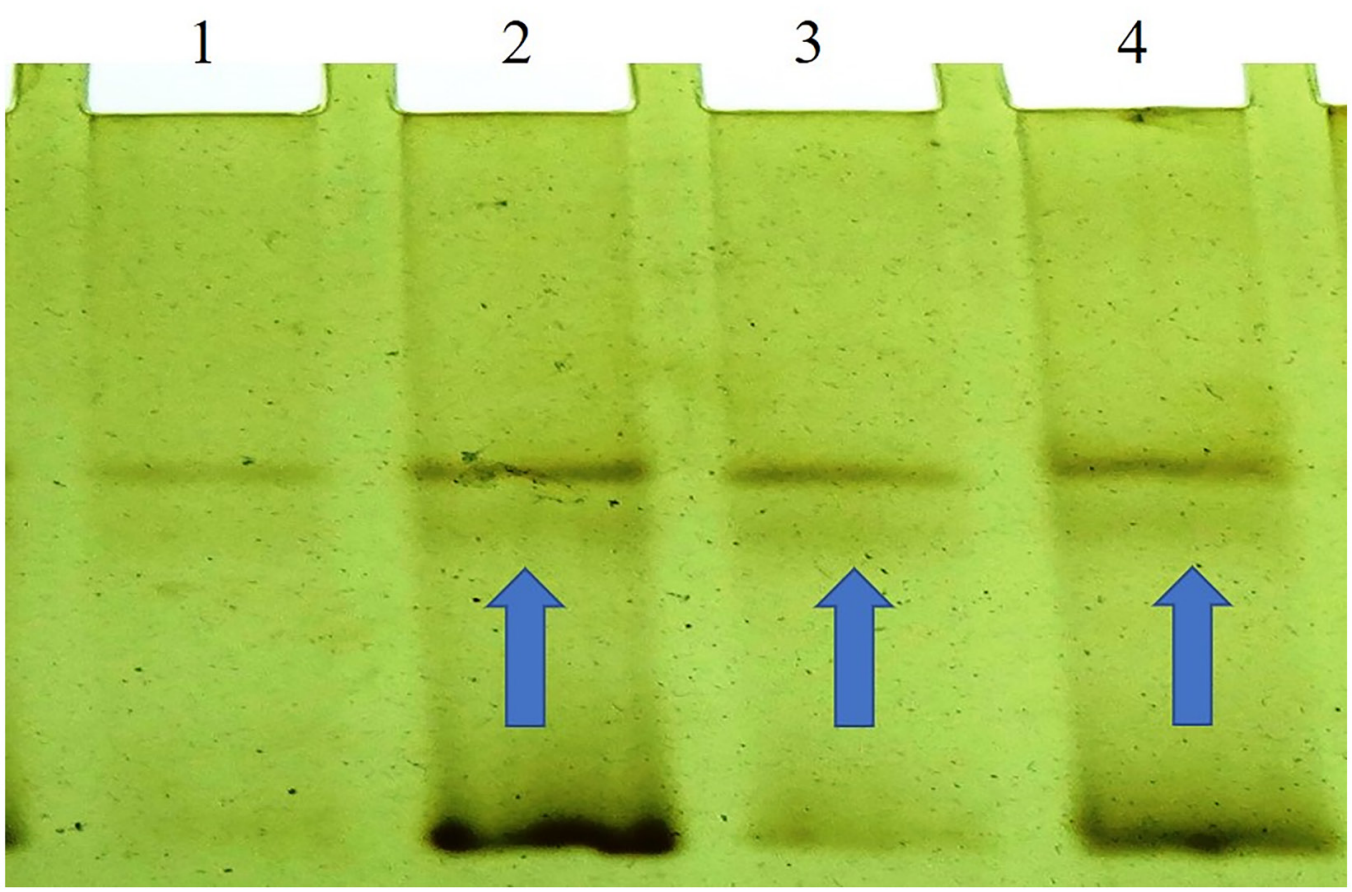
SSCP (non-radio-active) analysis of the BORIS gene which shows a variation or shifts in the band at Lane 2, 3, and 4 of the breast tumor samples.

**Figure 3:**
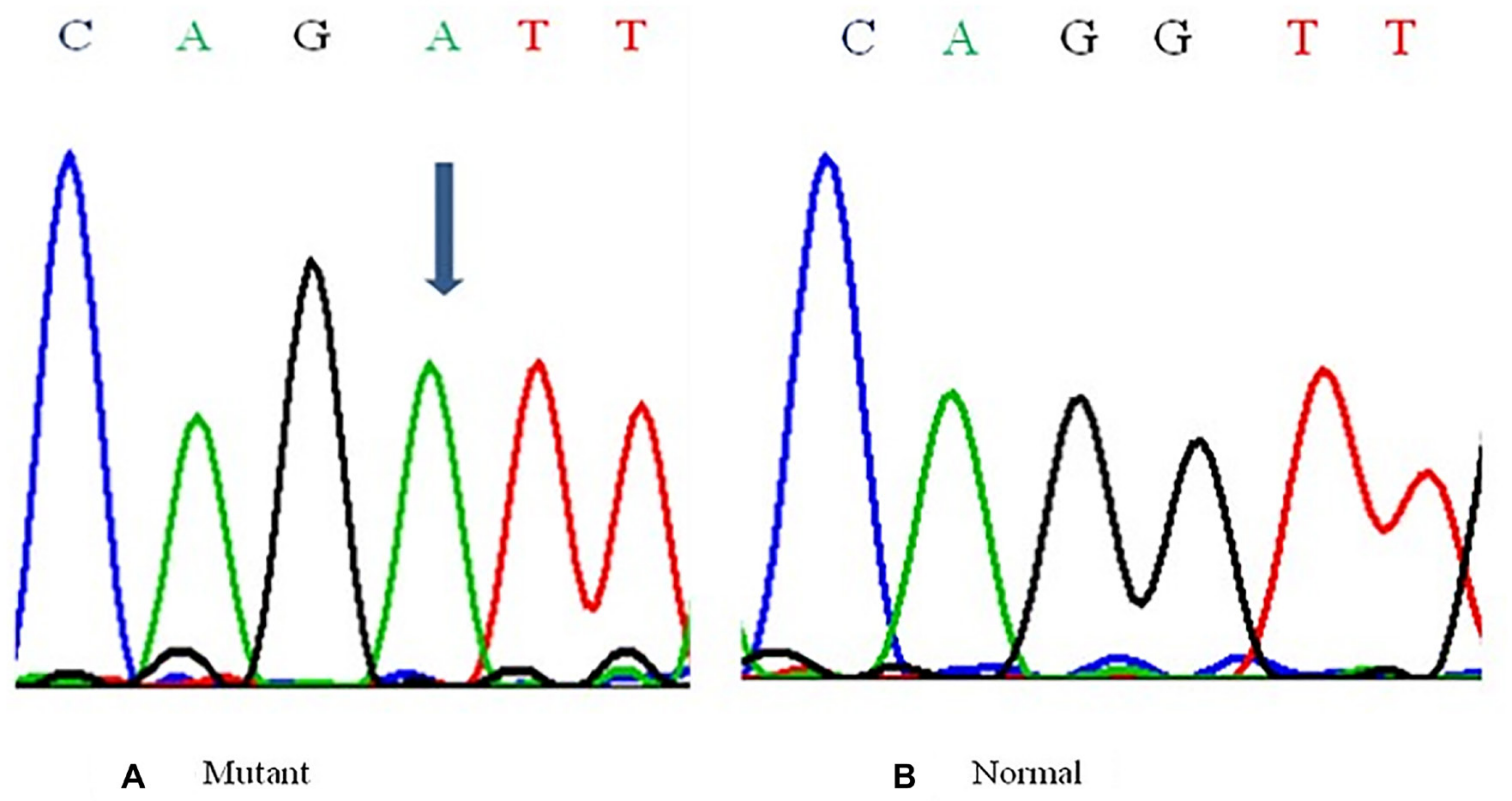
Representative Partial electropherograms of Mutant (**A**) (shown by arrow) with Normal (**B**) adjacent forms for the BORIS gene showing G>A transition.

The BORIS gene amplified PCR product of 352 bp (Figure 1) was electrophorized and a change or shift of single-strand DNA bands was analyzed in comparison to that of wild type for hotspot exons. Samples that showed alteration were identified as SSCP positive (Figure 2) and then were DNA sequenced to confirm the mutation and its types (Figure 3). The mutations found were missense (transition mutation) and is present in codon 329 leading to amino acid change Valine to Isoleucine (Val>Ile) and the base changes were GTT>ATT (G>A). A total of 25 (25/155, 16.12%) samples showed mutations in the exon 5 of the BORIS gene. The mutations found were a missense (Val>Ile) in the coding region harbored by twenty-five breast cancer cases (Figure 3).

The mutations found were exclusively associated with the breast tumor samples and were not present in the normal adjacent tissue sample. In the relationships between mutations of the BORIS gene and several clinicopathological variables a significant association was observed with the patients’ clinical stage (*p* = 0.010), nodal status (*p* = 0.013), ER expression (*p* = 0.008), PR (*p* = 0.039) expression and menopausal status (*p* = 0.023), (Table 1). However, the observed association with other pathological parameters like age (*p* = 0.299), histological status (*p* = 0.475), tumor size (*p* = 0.400), histological grade (*p* = 0.254), and Her2/Neu (*p* = 0.425) fail to show the level of significance statistically (Table 1).

**Table 1:** Correlation between mutations of the human BORIS gene with clinicopathological variables of breast cancer patients

Variables		No. of cases (*n* = 155)	Mutations	Mutation rate (%)	χ^2^ value	*P*-value
**Age**	>50	80	16	20.0	1.32	0.124
	≤50	75	09	12.0		
**Menopausal status**	Pre-menopausal status	70	06	8.57	3.95	0.023^*^
	Post-menopausal status	85	19	22.35		
**Histological status**	Invasive ductal carcinoma (IDC)	153	25	16.33	0.326	0.284
	Invasive lobular carcinoma (ILC)	02	00	00		
**Tumor Size**	≤2 cm	65	07	10.76	0.093	0.400
	≥2 cm	90	18	20.00		
**Histological Grade**	Poor differentiation (PD)	40	05	12.50	1.146	0.563
	Moderate differentiation (MD)	69	14	20.28		
	Wide differentiation (WD)	46	06	13.04		
**Clinical Stage TNM**	Stage II (a + b)	73	18	24.66	5.341	0.010^*^
	Stage III (a + b) + IV	82	07	08.54		
**Nodal Status**	Positive	81	19	23.46	4.915	0.013^*^
	Negative	74	06	08.10		
**Estrogen Receptor (ER) status**	Positive	72	18	25.00	05.621	0.008^*^
	Negative	83	07	08.43		
**Progesterone Receptor (PR) status**	Positive	66	06	09.09	3.079	0.039^*^
	Negative	89	19	21.35		
**Her2/Neu**	Positive	69	12	17.39	0.105	0.372
	Negative	86	13	15.11		

^*^ *P*-value < 0.05 was considered significant.

### BORIS expression and clinico-pathological features

**Figure 4:**
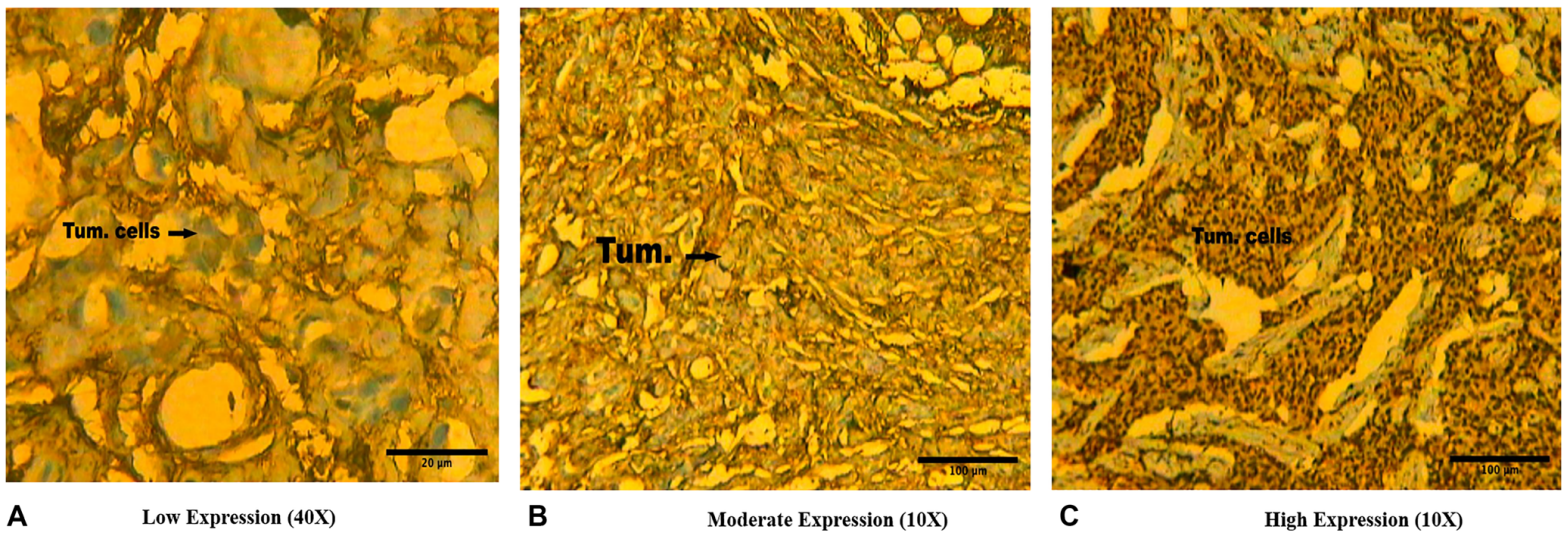
Representative immunohistochemical slides showing (**A**) Low protein expression (+) (scale bar 20 μm), (**B**) Moderate protein expression (++) (scale bar 100 μm), and (**C**) High protein expression (+++) (scale bar 100 μm) of BORIS protein in Indian female breast cancer cases.

The images of immunohistochemical slides seen at 10X and 40X magnifications are shown in Figure 4. Of the total 155 cases of breast cancer, 32 cases (20.64%) showed low/or nil protein expression (+), 53 cases (34.19%) showing moderate (++) expression and in 70 cases (45.17%) showed high (+++) expression of BORIS (Table 2, Figure 4). The significant association was observed between expression of BORIS protein with clinical stage (*p* = 0.013), nodal status (*p* = 0.049), ER expression (*p* = 0.039) and PR expression (*p* = 0.027) (Table 3). The correlation was found not significant with other features like age (*p* = 0.424), menopausal status (*p* = 0.679), histological status (*p* = 0.568), tumor size (*p* = 0.661), histological grade (*p* = 0.752) and Her2/Neu expression (*p* = 0.754) (Table 3).

**Table 2:** Profile of BORIS gene expression by immunohistochemistry

BORIS gene expression		
High (+++) expression	70/155	45.17%
Moderate (++) expression	53/155	34.19%
Low (+) expression	32/155	20.64%

**Table 3:** BORIS protein expression (IHC) pattern and effect in female breast cancer cases from India

Variables		No. of cases (*n* = 155)	Low	Normal	High	χ^2^ value	*P*-value
**Age**	>50	80	21	19	40	2.795	0.424
	≤50	75	17	27	31		
**Menopausal status**	Pre-menopausal status	70	21	20	29	1.513	0.679
	Post-menopausal status	85	32	18	35		
**Histological status**	Invasive ductal carcinoma (IDC)	150	34	40	76	2.021	0.568
	Invasive lobular carcinoma (ILC)	05	00	01	04		
**Tumor Size**	≤2 cm	65	09	12	44	1.592	0.661
	≥2 cm	90	17	21	52		
**Histological Grade**	Poor differentiation (PD)	40	10	14	16	3.435	0.752
	Moderate differentiation (MD)	69	12	17	40		
	Wide differentiation (WD)	46	10	14	22		
**Clinical Stage TNM**	Stage II (a + b)	73	16	17	40	10.693	0.013^*^
	Stage III (a + b) + IV	82	35	22	25		
**Nodal Status**	Positive	81	33	20	28	7.821	0.049^*^
	Negative	74	16	18	40		
**Estrogen Receptor (ER) status**	Positive	72	14	17	41	8.332	0.039^*^
	Negative	83	30	24	29		
**Progesterone Receptor (PR) status**	Positive (+ve)	66	30	14	22	9.161	0.027^*^
	Negative (−ve)	89	20	27	42		
**Her2/Neu**	Positive (+ve)	69	20	19	30	1.195	0.754
	Negative (−ve)	86	32	20	34		

^*^ *p*-value < 0.05 was considered significant.

### Correlation between BORIS mutations and expression in breast cancer cases

The observed mutations in the cases of breast cancer were analysed with the BORIS protein expression to draw the active role of BORIS in the patients of breast cancer. The association found between the mutations of BORIS and its expression was significantly assessed in high level (+++) category of protein expression (*p* = 0.017). Therefore, the association in the category of moderate (++) (*p* = 0.079) and low (+) level (*p* = 0.155) protein expression was not statistically significant (Table 4).

**Table 4:** Association of mutation and protein expression (IHC) of BORIS gene in female breast cancer cases from India

Level of protein expression	No. of cases (*n* = 155)	Mutation (25/155 = 16.12%)	*χ*^2^	*p*-value
High (+++) expression	70/155 (45.17%)	17/155 (10.97%)	4.497	0.017^*^
Moderate (++) expression	53/155 (34.19%)	05/155 (3.22%)	1.986	0.079
Low (+) expression	32/155 (20.64%)	03/155 (1.93%)	1.027	0.155

^*^ *p*-value < 0.05 was considered significant.

## DISCUSSION

BORIS, a family member of CTA gene, and its transcription in various cancer cells and tumors is activated abnormally [11, 14–16, 27, 47]. Abnormally, BORIS RNA and protein expression levels affected by methylations of DNA, correlated with tumors size and degree of malignancy, alternatively the knockdown of BORIS induced apoptosis in tumorous cells [27]. BORIS and CTCF do not compete although they are having the same recognition sites in normal somatic cells. However, BORIS expression in negative cells of BORIS does not interfere only with the normal functioning like growth inhibition of CTCF, but also lead to dysfunctions of cells, leading to the process of tumorigenesis due to the binding of BORIS/CTCF gene family competitively [37]. Recently, studies suggested that ectopic expression of BORIS activates cancer CTAs and components of cancer relevant signaling pathways [56]. BORIS is the paralogue of CTCF and is speculated to compete with CTCF to induce the expression of the oncogene [11, 30, 57]. Although BORIS is not observed in most cancers, but their aberrant expression was reported in breast cancer cells [17]. Therefore, it is indicated that BORIS expression was crucial in modulating cell viability in cancer cells in addition to CTCF regulation [58].

In this study, the important hotspot coding region of BORIS gene has screened for mutational analysis by the molecular technique PCR-SSCP in 155 female patients of breast cancer and found 25 (25/155, 16.12%) missense mutations. The identified mutations located at codon 329 lead to Valine>Isoleucine (G>A). Importantly, the sequence changes of BORIS were associated exclusively with cells of isolated breast cancer, and not found in adjacent normal tissues. The transition for G>A at nucleotide position 6267 led to a change from Valine to Isoleucine in breast cancer patients, and this amino acid residue is evolutionary conserved, and this variant acts as a risk factor for tumor progression. Moreover, our data exhibited the altered expression profiles of BORIS which may be due to the result of potential mutation(s) in the BORIS exonic region and thus can contribute in the progression of breast cancer as shown in an early study [17]. The analysis of potential relationship with the patient’s menopausal status of patient’s ages, histological types and grades, size of tumors and stages, metastases of lymph node, expression status of ER & PR and Her2/neu expression. To elucidate the role of mutation(s) in BORIS gene in the progression of breast cancer revealed a significant association of BORIS mutation and patients’ clinical stage (*p* = 0.010), nodal status (*p* = 0.013), ER (*p* = 0.008), PR (*p* = 0.039) expression and menopausal status (*p* = 0.023) (Table 1). The significant association of mutations with the clinical parameters further emphasizes the link between BORIS and breast cancer progression [59]. The association was observed with other clinical features like patients’ ages, tumor sizes, histological types, and grades, and Her2/neu amplifications were statistically not significant. The HER2/neu positive breast cancer subtype lacks ERα and PR expression with amplified HER2/neu expression and clinically has a worse course as compared to the luminal breast cancer subtypes. However, in the HER2/neu positive sub-types of breast tumors, there is a high degree of heterogeneity found [60].

BORIS protein is present or absent at low levels in non-cancerous cells and tissues, but it is present at higher levels in all cancer cell lines and tumors, indicating that BORIS might be used as a potential cancer biomarker [47, 61, 62]. BORIS protein expression analysis was performed to explore the possible role of BORIS in female breast cancer cases and also to define their biomarker property. The immunohistochemical staining revealed that the levels of BORIS protein were significantly higher in all breast tumors compared with normal. An early study showed moderate to strong cytoplasmic staining of the BORIS protein [17]. More than 80% of the cases included in our study showed a moderate or high BORIS expression which was significantly linked with the clinical stage (*p* = 0.013), nodal status (*p* = 0.049), ER expression (*p* = 0.039), and PR expression (*p* = 0.027) (Table 3) which play an important role in the progression of carcinoma of the breast. The correlation was found not significant with other clinical features like age (*p* = 0.424), menopausal status (*p* = 0.679), histological status (*p* = 0.568), tumor size (*p* = 0.661), histological grade (*p* = 0.752) and Her2/Neu expression (*p* = 0.754) (Table 3) suggesting the involvement of detected mutations in expression and altered formations of the protein contributing to the onset and progression of breast cancer which is in agreement with the findings of an early study [59].

The altered expression profiles of BORIS showing mainly cytoplasmic expression in the current study are in agreement with the earlier studies [17]. Mutation and protein expression analysis were combined and found a significant association in the category of high (+++) level of BORIS expression showing greater number of mutations (Table 4). Hence it seems that the progression of breast cancer was found to be related with higher level of BORIS expression and greater number of mutations with disease progression. Therefore, it is speculated that BORIS may act as a potential biomarker in breast cancer progression [13].

BORIS is involved in numerous cellular processes [37, 63] many of which are altered during carcinogenesis, the loss of BORIS expression also suppresses the Warburg effect and enhance the growth of cells of breast cancer [64]. The properties of BORIS gene as transcription regulator control the expression of promoters of PR [65] and ER [66]. It also activates both promoters, therefore suggesting that BORIS may play as a positive transcription regulator of both the genes. The Clinical significance of BORIS expression was assessed in breast tumors and also it is observed that high level of BORIS expression is associated with high level of ER and PR expression. The estrogen and progesterone are one of the important hormones in the development of mammary resulting in cell growth stimulation, their proliferation and differentiation in humans [67–69]. Therefore, it is demonstrated that both hormones promote the processes of tumorigenesis of breast [70, 71].

The BORIS appearance during the process of gametogenesis and tumorigenesis hypothesizes that the programme of gametogenetic induction in somatic cells may also be correlated with development of tumour cells [72, 73]. Therefore, it is observed that BORIS functions as a regulator of upstream for various CTA gene [14, 39]. As per recent reports it is revealed that the mechanisms of epigenetic and genetic factors are implicated in BORIS activation processes. The functions of p53, CTCF and DNA methylation perform an important role in deregulation of gene promoters of BORIS [74]. The utilization of various promoters selectively [74] and their splicing alternatively leads to contribute the expression of BORIS regulation in different types of cells. The role of BORIS gene association in malignant and immortalized cells shows BORIS can perform an active role in maintenance and establishment of proliferation of cells and also act as responsible for genes activator in particular tissues, like gene expression of ER and PR in glands of mammary cells.

Thus, BORIS can also stimulate the production of ER and PR expression that indicates towards possible BORIS involvement in the establishment, maintenance, and progression of tumours of the breast tissues. However further large-scale population size study which we are planning is required for the establishment of BORIS as a prognostic biomarker and to be called as oncogene.

## MATERIALS AND METHODS

### Biological sample collection

A total number of 155 samples of female breast tumour tissues and equal number of adjacent normal tissues (measuring from 5 to 10 mm) which were not tumor infiltrated were taken after confirmation by pathologist, from Surgical Oncology department, All India Institute of Medical Sciences (AIIMS) New Delhi, India. The inclusion criteria of sample collection from patients were specimens only from all types of the breast carcinomas were taken, specimen from breast cancer patients who have not received chemotherapy, and breast carcinomas of all the stages were included. The exclusion criteria of sample collection were patients who have undergone neoadjuvant therapy which were not included, also the patients with no gross tumor and other malignancies of the breast were not included in the study. The stages of breast cancer were determined by using the TNM staging system or American Joint Committee on Cancer (AJCC). The collected biopsies were put in formalin and phosphate buffer saline during the period of 2009 to 2014 and stored in −80°C for further use. The prior informed consent was filled after ethical approval from ethical committee of the institutes, AIIMS and Jamia Millia Islamia University, New Delhi, India.

### DNA isolation from tissues

The genomic DNA was extracted from tumor of breast and normal adjacent tissue samples by using DNA isolation Kit (Qiagen, USA). The Qiagen DNA isolation Kit is specially designed for purifying DNA from formalin-fixed, paraffin-embedded tissue sections. The kit uses special lysis conditions to release DNA from tissue sections and to overcome inhibitory effects caused by formalin crosslinking of nucleic acids. The kit uses QIA amp Min Elute spin columns for purification of high-quality DNA in small volumes. The purification of DNA using the QIA amp DNA FFPE Tissue Kit can be automated on the QIA cube Connect.

The isolation of DNA from tumour tissues consists of six steps: first remove the paraffin, after that lyse the samples, then heat, and bind, wash, and elute. After samples lysis, the simple QIA amp DNA FFPE Tissue procedure, which is highly suited for simultaneous processing of multiple samples, yields pure DNA in less than 30 minutes. The isolated genomic DNAs quality was assessed by 2% agarose gel electrophoresis followed by UV spectrophotometer.

### PCR-SSCP analysis

A typical PCR amplification was performed in a 25 μl reaction volume (10 mM Tris–HCl pH 8.4, 50 mM KCl, 1.5 mM MgCl2, 200 l M of each dNTPs, 100–500 ng of tumor DNA, 0.5 U Taq polymerase and 5 pmol of each oligonucleotide primer) along with negative controls. The BORIS gene hot spot exon 5 was probed for mutation analysis by PCR with the primer sequences 5′TCTCACATGCATCTGTGGTA3′ (forward) and 5′TGGAGTAACTTGTACAGCAG3′ (reverse). The PCR products were electrophoresed by using ethidium bromide-stained 3% agarose gel in 1X TAE with 100-bp molecular weight marker as reference to check the presence of 352 bp amplified DNA sequence of interest using the Gel doc system (Bio-Rad). The DNA of high quality was screened for mutations in the BORIS gene by SSCP analysis performed according to the method described earlier by Orital et al., 1989. 10 μl of the resultant PCR product were mixed with 8 μl of formamide gel-loading buffer and heated at 95°C in a thermos-cycler for 5 min. Immediately it was chilled on ice for 15 min to restrict the DNA renaturing in double stranded form. Then the product was loaded in 1X TEMED gel, electrophoresed for 16–18 hours at a constant voltage at optimal room temperature. The gel was stained with silver nitrate in the dark room. Alteration or changes in the band movement were recorded as SSCP positive.

The appearance of 352 base pair amplicon of PCR product (Figure 1), was identified by applying the Quantity One Software (Laboratories of Bio-Rad, CA, USA). Alteration in electrophoretic mobility of any band as earlier given by [75], the purified PCR product was assayed. Silver staining was carried out as earlier described [76, 77]. The labeled silver-stained gel was scanned for analysis of computer imaging and documentation. The SSCP positive samples (Figure 2) were identified by single-strand bands of DNA of tumor and normal tissues samples were compared.

### DNA sequencing

The samples which showed variant band-shifts in SSCP were re-amplified for sequencing. The PCR products were extracted using a Gel Extraction Kit (Qiagen) and then sequenced using an ABI prism 310 Automated Sequencer. Before sequencing, the PCR products were purified using ammonium acetate/ethanol precipitation method. The cycle sequencing of the purified products was performed using BIG dye Terminator Cycle Sequencing Ready Reaction Mix with Amplitaq DNA Polymerase FS, on the Gene AMP PCR 9700. The PCR conditions were set as follows: 96°C for 10 sec, 50°C for 5 sec, and 60°C for 4 min for 25 cycles. After cycle sequencing, extension products were purified to remove the unincorporated dye-labeled terminators using the ethanol/sodium acetate precipitation method. The air-dried labeled purified products were resuspended in 20 ml template suppressor reagent, chilled on ice and loaded on the 310 sequencers. The sequences were analyzed using Sequencing Analysis Software 3.4.1. on a Mac OS 9.1.

The SSCP positive DNA samples were sequenced by Sci-Genome, laboratory located at Cochin, India and further double sequencing processes were carried out to exclude any contamination and/or PCR artifacts for confirmation of mutations and its type.

### Methods of nodal status, ER & PR and Her2/neu expression

#### Nodal expression

The expression of gene signature has been used to assess the lymph node metastasis status of breast cancer. Additionally, the nucleosome footprint of cell-free DNA (cfDNA) carries gene expression information of its original tissues, therefore, it may be used to evaluate the axillary lymph node status in breast cancer.

#### ER & PR expression

For the expression study of ER & PR, the most commonly used method is immunohistochemistry (IHC) to test tumor tissues for estrogen and progesterone receptors. IHC testing can detect estrogen and progesterone receptors in cancer cells from a sample of tumor tissues.

#### Her2/neu expression

For the analysis of Her2/neu expression the most widely used method, is either an immunohistochemistry (IHC) test or fluorescence *in situ* hybridization (FISH) test used to find out the cancer cells that have a high level of the HER2 protein. Based on the score, the expression will be determined.

Accordingly, If the score is 2+, it is considered borderline. A score of 3+ is considered HER2-positive. However, if the IHC test results are found at the borderline, then it is likely that a FISH test will be performed on a sample of the cancer tissue to determine whether the cancer is HER2-positive.

### Immunohistochemistry

#### Construction of tissue microarray

Before performing immunohistochemistry, tissues were checked and stained with hematoxylin and eosin (H&E) for analyzing proper morphological characteristic of the tissue samples. The core tissue biopsies were collected in formalin and moved on paraffin embedded slides. For improvement of adhesion processes between paraffin and tissues, mild incubation was given at the temperature of 55°C.

#### Immunohistochemical analysis

To assess the BORIS protein expression, the immunohistochemical staining processes were performed by applying BORIS anti-human polyclonal antibody (Bio Vision, USA, Catalog # 3888-100) [78].

The paraffin slides were dipped three times into xylene, 5 min in each to deparaffinize, then into alcohol 100, 95, 85, and 75% successively 2–3 min to rehydrate the tissue and heat treated in citrate buffer solution (pH 6.0) at 72°C for 10 min for antigen retrieval.

For retrieval of antigen the cancer tissue samples of breast were cut into section of 2 to 4 μm, which is attached with coated slides of poly-L-lysine and therefore treated in the solution of xylene, alcohol and with heat treatment. In order to block the endogenous peroxidase activity, slides were dipped in hydrogen peroxide (10 ml of 30% H2O2) for 30 min. Finally, incubated slides were treated with anti-BORIS antibody and were developed by applying the detection kit streptavidin Horse-reddish peroxidase (Bio Vision USA). The distribution of BORIS positivity was scored according to Beck et al. 1995. The scoring of slides was as in the category of Low or no (+) expression of protein, Moderate (++) expression and High (+++) BORIS protein expression.

The scale bar in the IHC Figure 4 was measured by using the Image J-software. By using Olympus BX 50, Tokyo the number and cells distribution were represented among the cases and controls groups was performed.

### Statistical analysis

The correlation of BORIS mutations and their protein expression was assessed with several clinicopathological variables, the Chi-square (χ^2^) test was performed by using Graph-Pad Prism 6.0. The probability value (*p*-value) ≤ 0.05 was considered significant.

## Abbreviations

BORIS: Brother of the regulator of imprinted sites
CTA: Cancer testis antigen
CTCF: Chromatin transcription factor
PCR: Polymerase chain reaction
PCR-SSCP: Polymerase chain reaction-single standard confirmation polymorphisms
Val: Valine
Ile: Isoleucine
ER: Estrogen receptor
PR: Progesterone receptor
IDC: Invasive ductal carcinoma
ILC: Invasive lobular carcinoma
PD: Poor differentiation
MD: Moderate differentiation
WD: Wide differentiation
AJCC: American Joint Commission on Cancer
IHC: Immunohistochemistry
FISH: Fluorescence in situ hybridization
H & E: Hematoxylin and eosin
χ^2^: Chi-square
*p*-value: Probability value
